# Coercivity Modulation in Fe–Cu Pseudo‐Ordered Porous Thin Films Controlled by an Applied Voltage: A Sustainable, Energy‐Efficient Approach to Magnetoelectrically Driven Materials

**DOI:** 10.1002/advs.201800499

**Published:** 2018-06-20

**Authors:** Evangelia Dislaki, Shauna Robbennolt, Mariano Campoy‐Quiles, Josep Nogués, Eva Pellicer, Jordi Sort

**Affiliations:** ^1^ Departament de Física Universitat Autònoma de Barcelona (UAB) E‐08193 Bellaterra Spain; ^2^ Institut de Ciència de Materials de Barcelona (ICMAB‐CSIC) Campus UAB E‐08193 Bellaterra Spain; ^3^ Catalan Institute of Nanoscience and Nanotechnology (ICN2) CSIC and the BIST Campus UAB E‐08193 Bellaterra Spain; ^4^ Institució Catalana de Recerca i Estudis Avançats (ICREA) Passeig Lluís Companys 23 E‐08010 Barcelona Spain; ^5^ Departament de Física Universitat Autònoma de Barcelona (UAB) E‐08193 Bellaterra Spain; ^6^ Institució Catalana de Recerca i Estudis Avançats (ICREA) Passeig Lluís Companys 23 E‐08010 Barcelona Spain

**Keywords:** coercivity, colloidal templating, electrodeposition, hierarchical porosity, magnetoelectric effects, voltage‐driven effects

## Abstract

Fe–Cu films with pseudo‐ordered, hierarchical porosity are prepared by a simple, two‐step procedure that combines colloidal templating (using sub‐micrometer‐sized polystyrene spheres) with electrodeposition. The porosity degree of these films, estimated by ellipsometry measurements, is as high as 65%. The resulting magnetic properties can be controlled at room temperature using an applied electric field generated through an electric double layer in an anhydrous electrolyte. This material shows a remarkable 25% voltage‐driven coercivity reduction upon application of negative voltages, with excellent reversibility when a positive voltage is applied, and a short recovery time. The pronounced reduction of coercivity is mainly ascribed to electrostatic charge accumulation at the surface of the porous alloy, which occurs over a large fraction of the electrodeposited material due to its high surface‐area‐to‐volume ratio. The emergence of a hierarchical porosity is found to be crucial because it promotes the infiltration of the electrolyte into the structure of the film. The observed effects make this material a promising candidate to boost energy efficiency in magnetoelectrically actuated devices.

## 1 Introduction

The potential toward voltage manipulation of magnetism is currently a research topic of tremendous interest due to its numerous prospective applications in areas such as magnetic data storage or spin‐based electronics.^1^ Despite the short screening length of metals, which was previously thought to be a limiting factor for the electric field effect,^2^ the magnetic properties of ferromagnetic films, such as saturation magnetization, coercivity, magnetoresistance, and magnetic anisotropy have been successfully manipulated using electrical methods including voltage control.^3^, ^4^, ^5^, ^6^, ^7^, ^8^, ^9^, ^10^, ^11^, ^12^ In particular, the reduction of coercivity using electric fields is an appealing approach for minimizing power dissipation during magnetic actuation. More specifically, in order to switch the magnetization, a magnetic field larger than the coercivity needs to be externally applied. Electric currents are needed to generate such magnetic fields (or to switch magnetization by spin‐torque effect). Since flowing electric currents are associated with Joule heating power dissipation, a reduction of the coercivity with DC voltages can drastically boost the energy efficiency in magnetically actuated devices. Voltage control of magnetism can be achieved through several mechanisms. These can be divided in five categories: (i) carrier accumulation, (ii) strain, (iii) exchange coupling, (iv) orbital reconstruction, and (v) electrochemical effect.^1^ The possible coupling between different magnetoelectric effects when the film thickness is in the range of several nanometers and the determination of the dominant factor are crucial issues requiring further investigation.^1^

In the case of ferromagnetic metals, carrier accumulation and relaxation is often driving the change in magnetism.^13^, ^14^ In bulk metals, the strong screening effect prevents penetration of the electric field throughout the entire metal. However, in the case of ultrathin metallic films with a high surface‐to‐volume ratio, a large electric field can be used to control carrier density, and thus magnetism. The use of liquid electrolytes has been shown to promote a large electric field owing to the very small thickness of the electric double layer (EDL) (typically <1 nm), which enables adjustment of the magnetic properties to a considerable extent.^2^ However, the need to use ultrathin magnetic layers is hampering the development of practical devices based on these effects. Previously, with respect to thicker films, this phenomenon had only been achieved when mediated by ion migration (magnetoionics)^11^ or by producing nanoporous alloys through highly sensitive and complex means such as micelle‐assisted electrodeposition in order to bypass the requirement of an ultrathin film.^3^ For this reason, a high surface area obtained by porosity was deemed a critical factor in attaining the desired magnetoelectric effect in films with a thickness of hundreds of nanometers. Porous metallic materials have been prepared in literature through the use of hydrogen bubble dynamic template,^15^, ^16^ block copolymers,^17^, ^18^, ^19^ surfactant micelles,^20^ and hard templates.^21^, ^22^ Among these, colloidal templating is a particularly advantageous method since it entails a swift and straightforward approach which, in conjunction with the electrodeposition technique, allows for the creation of a multilayered 3D architecture with controllable pore size. Moreover, the close packing of the colloidal spheres is conducive to the development of exceptionally thin pore walls.

In this work, hierarchically porous thin Fe–Cu films have been prepared by electrodeposition through colloidal templates onto metallized substrates. Three macropore sizes (in the 200–500 nm range) were achieved through the selection of distinct colloidal sphere diameters. The close packing of the spheres, which was achieved by electrophoretic deposition, enabled the formation of ultrathin pore walls, which were found to exhibit an additional inherent nanoporosity according to ellipsometry measurements, thereby rendering a truly hierarchically porous alloy structure. The macropores are dispersed forming ordered or pseudo‐ordered areas of ≈1 µm^2^. Importantly, the dual porosity leads to a high surface‐to‐volume ratio, which combined with the ultrathin pore walls, yields an excellent material for carrier density modulation^2^ despite an overall film thickness of hundreds of nanometers. In fact, reversible coercivity (*H*_c_) reduction in the range of 21.6–25.3% was observed in all cases. Morphological, chemical, and structural analyses were carried out to correlate the structure–morphology with the underlying mechanisms of the voltage control of magnetism.

## 2 Results and Discussion

### 2.1 Sample Morphology and Porosity Assessment

**Figure 1:**
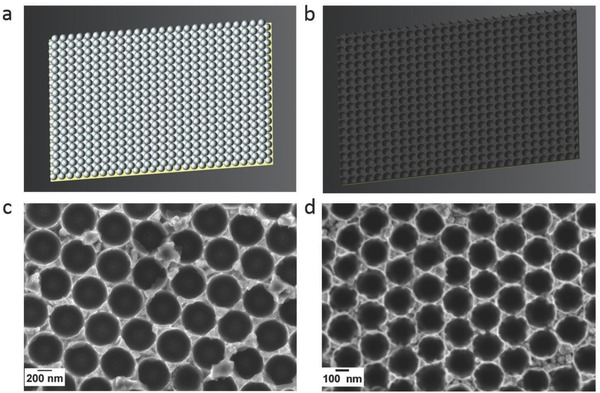
Schematic drawings of a) the assembly of PS spheres onto the metallized Si substrate and b) the electrodeposited Fe–Cu film after removal of the nanospheres. FESEM images of resulting Fe–Cu films of c) 500 and d) 200 nm pore size.

**Figure 2:**
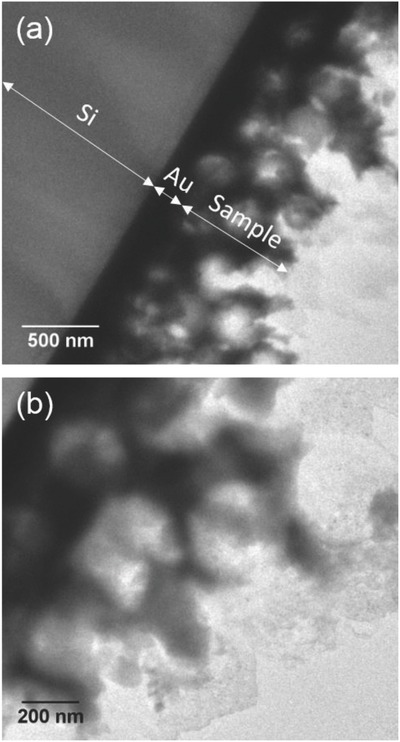
Transmission electron microscopy (TEM) images of a cross section of a porous Fe–Cu electrodeposited film (obtained using colloidal templating with spheres of 350 nm), revealing that the walls of the pores are not fully dense.

Schematic drawings of the colloidal templating process are given in **Figure** **1**a,b. Representative field emission scanning electron microscope (FESEM) images of the porous Fe–Cu films obtained after removal of the polystyrene (PS) spheres are shown in Figure 1c,d. In this case, a monolayer assembly of PS spheres was targeted. It can be clearly observed that the pore walls are extremely narrow and, in some locations, they are even below 10 nm. In addition, transmission electron microscopy (TEM) imaging performed on a multilayer sample reveals that the pore walls are, in turn, porous (as shown in **Figure** **2**). We can, therefore, consider that the pore walls are equivalent to ultrathin films for the purpose of assessing eventual voltage‐driven magnetoelectric effects. Also, it should be noted that, while the films were electroplated under the same conditions, the composition for the 500 and 350 nm pore dimensions was ≈85 at% Fe while for the smallest sphere size of 200 nm the atomic percentage of Fe fell to 75 at%. This is on account of the formation of expectedly narrower interstices during packing of the smallest 200 nm diameter PS spheres. Since deposition of Cu is mass transport controlled,^23^ Cu cations can probably infiltrate the spaces between the spheres in a more facile manner, thus resulting in compositions slightly more depleted in Fe. Another reason could be that the potential inside the spaces was positively shifting from the overpotential for Fe reduction as the cavity size decreased.

**Figure 3:**
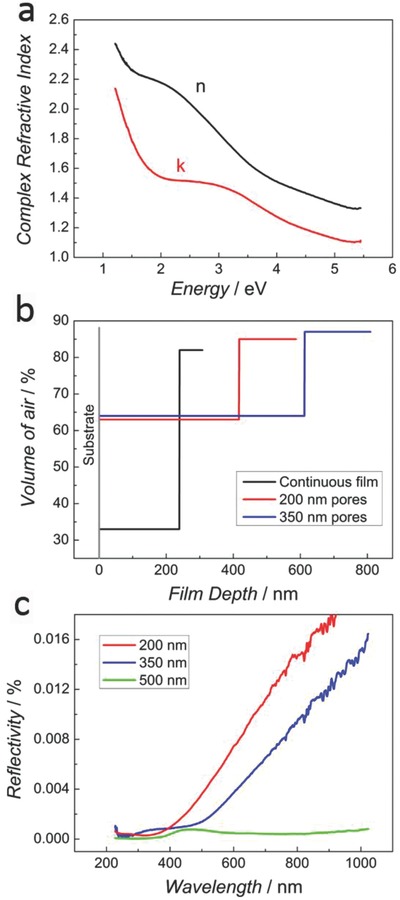
Results from ellipsometry measurements related to a) optical properties derived from sputtered sample (compact reference), b) volume of air contained in electroplated continuous film compared to porous samples when modeling the films as two sublayers with different void content, and (c) reflectivity of porous deposits at an incident angle of 30°.

To evaluate the average degree of porosity inherent in the material, we first carried out spectroscopic ellipsometry on the reference electroplated (continuous, i.e., unpatterned and multilayer samples of three macropore sizes) and sputtered samples of similar composition. The optical properties for the sputtered sample are shown in **Figure** **3**a. The refractive index of this sample was then used as a reference to model the data for the electroplated samples and infer the average air content by taking the effective medium approximation of the optical properties via the Bruggeman model.^24^ For the electroplated samples, two clear observations could be made. First, all electroplated samples, including the continuous case, exhibit a lower density compared to the sputtered sample, as the models contain large amounts of air (i.e., voids). Since the crystalline structure is the same, the lower density is here ascribed mainly to some degree of nanoscale porosity. The electroplated continuous film contained about a 33% volume fraction of air compared to the sputtered reference sample. This result from ellipsometry can be further supported by high magnification FESEM images (not shown) which reveal the existence of disperse nanopores. In comparison, the use of colloidal templating increased the porosity value to around 65%. This is compatible with a hierarchical distribution of pores.

The second observation is that the air distribution is not homogeneous throughout the depth of the films, as the outer surface appears to be much more porous than the inner layers. Approximated depth profiles of air within the films are shown in Figure 3b for a simple description of the layer as two sublayers with different void fractions (see the Experimental Section for details). In this graph, the zero in the *X*‐axis corresponds to the substrate, and the curves extend through the depth of the film until the total thickness is reached (where each horizontal line finishes abruptly), thus marking the surface with air. This dual porosity results in a gradual variation of the index of refraction, akin to an antireflective coating, which results in a very low reflectivity in these materials (Figure 3c). The reflectivity becomes lower with increasing pore size, as the refractive index decreases (reducing index contrast with air) and scattering increases due to pore dimensions which become comparable to the wavelength of light.

### 2.2 Magnetoelectric Measurements and Coercivity Reduction

**Figure 4:**
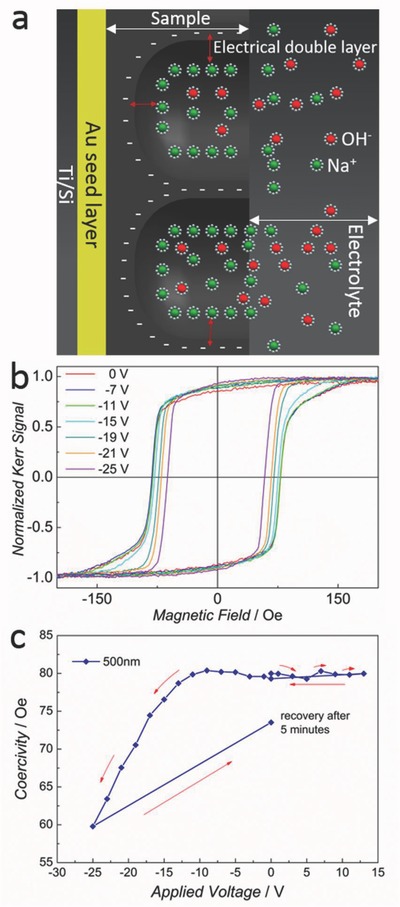
a) Schematic drawing to illustrate the formation of the electrical double layer at pore walls/electrolyte interface. b) Hysteresis loops captured for different values of applied voltage and c) voltage dependence of coercivity in the range of +13 to –25 V for the 500 nm pore sample.

The magnetoelectric measurements were performed with samples obtained using each distinct colloidal sphere size (with a monolayer thickness, i.e., resulting from plating between the interstices of a single layer of PS spheres, to reduce the lack of reflectivity issues), as well as with the sputtered and electroplated continuous Fe_85_Cu_15_ samples. The formation of an electrical double layer at the pore walls/electrolyte interface (as depicted in **Figure** **4**a) caused the existence of large electric fields upon application of external voltages.

**Figure 5:**
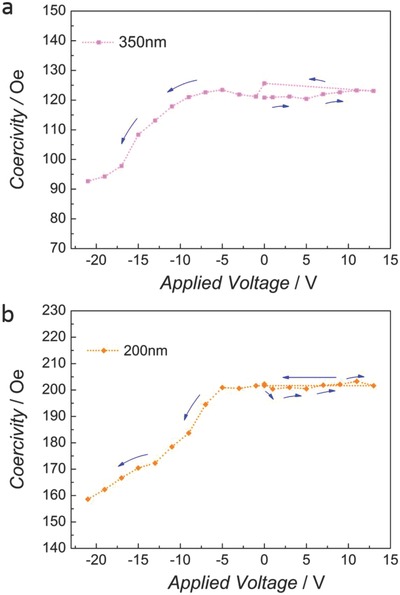
Dependence of the coercivity on the applied voltage for the electrodeposited Fe–Cu films grown using colloidal templated substrates, with spheres of a) 350 and b) 200 nm.

The protocol followed was to initially record a hysteresis loop at 0 V, then gradually increase the applied voltage in a stepwise manner up to +13 V and record the loops at each value of applied voltage. It was first observed that application of positive values of voltage does not lead to a substantial change in coercivity (Figure 4c). The coercivity remained the same even when applying voltages as high as +25 V. Subsequently, a waiting time of 20 min was introduced after which the sample was again measured at 0 V (with no appreciable changes with respect to the pristine as‐deposited sample). Next, the voltage was decreased in steps until reaching –25 V. A clear coercivity reduction was observed for the three porous samples, with ≈21.6, ≈23.3, and ≈25.3% decrease for the 200, 350, and 500 nm samples, respectively. These changes are considerably more pronounced than those observed in the seminal work by Weisheit et al. in ultrathin (nonporous) FePt and FePd films (where the maximum decrease in *H*_c_ was only 4.5%).^2^ The most pronounced *H*_c_ modulation from 0 to −25 V found for the 500 nm pore sample is illustrated in Figure 4b,c. **Figure** **5** shows the dependence of *H*_c_ on applied voltage for the 350 and 200 nm sphere diameters. Importantly, the Kerr amplitude (which is proportional to the saturation magnetization in a first approximation) does not show variations for different voltage values. As expected, for the sputtered, virtually pore‐free film, no *H*_c_ changes were observed with variation of the applied voltage. Finally, the electrodeposited continuous film showed only small fluctuations with an average coercivity value of ≈117 which indicates that the electrolyte is interacting mainly with the surface but could not easily get infiltrated into the film. Moreover, it is apparent that the inherent nanoporosity of the material is not sufficient to induce any sizable magnetoelectric effects in the absence of the well‐defined, ultrathin pore walls. Therefore, the results confirm that the porous structure created by colloidal templating is indeed essential for the magnetoelectric effect as it allows for the diffusion of the electrolyte throughout the entire porous network and leads to extremely thin pore walls which are, in a sense, equivalent to ultrathin films.

### 2.3 Investigation of Crystalline Structure and Surface Elemental Composition during Voltage Actuation

**Figure 6:**
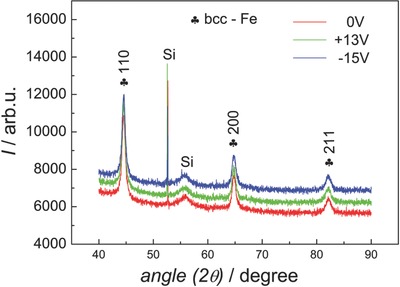
X‐ray diffractograms of a representative sample taken in as‐prepared condition and following negative and positive values of voltage applied.

Upon verification of the feasibility of the voltage control of magnetism for the hierarchically porous Fe–Cu deposits, further examination of their structural characteristics and surface composition was carried out with a special emphasis on identifying and tracking any eventual changes resulting from the application of voltage, which could contribute to the observed magnetoelectric effects, besides the inherent electric charge accumulation. The grazing incidence X‐ray diffraction (GIXRD) patterns of a representative multilayer sample of 350 nm macropore size in the as‐prepared state (i.e., before any voltage manipulation) as well as after subjecting it to DC voltages of +13 V for 20 min and –15 V for 20 min, are shown in **Figure** **6**. As can be readily discerned, there are no detectable shifts in the diffraction peaks for the large negative and positive voltage values (i.e., no changes in the cell parameter). In addition, no other peaks (e.g., stemming from oxidation or a phase transition) were visible either. It should be noted that all the porous films present the same peaks corresponding to body‐centered cubic (bcc) Fe and are, therefore, single phase. Typically, phase separation into bcc and face‐centered cubic (fcc) phases can occur in this range of compositions for electrodeposited Fe–Cu films with a thickness of several micrometers.^25^ However, it was confirmed that this was not the case for continuous films of 250–300 nm thickness as in our case (only peaks from bcc Fe were observed, not shown). Additionally, in the multilayer porous films, the formation of a single phase is promoted by the forced growth in the narrow interstices between the PS spheres. It has been previously shown that confining electrodeposition in narrow cavities close to or comparable to crystallite size can suppress phase separation.^23^ Thus, the GIXRD results clearly indicate that the films remain unaltered during the voltage application.

**Figure 7:**
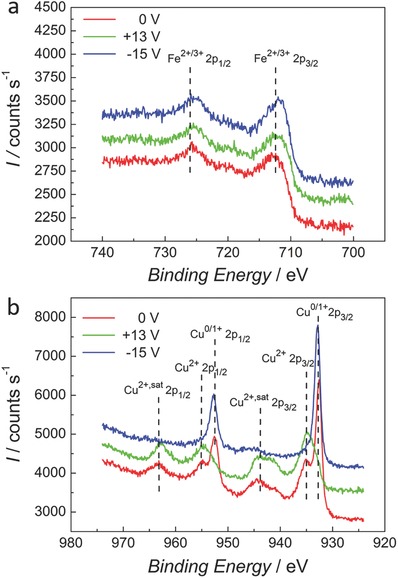
XPS detail spectra of a) Fe 2p and b) Cu 2p peaks of a chosen sample taken at as‐prepared conditions and after voltage modulation.

Subsequently, the chemical composition of the uppermost surface layer was examined using X‐ray photoelectron spectroscopy (XPS). This revealed the presence of mainly Fe and Cu oxides at the surface (i.e., a thin oxide passivation layer), which were not detectable by XRD as they are below the threshold detection limit. As seen in **Figure** **7**a for the sample of 350 nm pore size, there is no appreciable change in the oxidation state of Fe which presents mainly as a mixture of Fe^2+^ and Fe^3+^ oxidation states. Contrarily, as shown in Figure 7b there is a clear shift in the Cu oxidation states, where for +13 V there is an enhancement of the Cu^2+^ peak. When the negative voltage of –15 V is applied, the Cu^2+^ peak and corresponding satellites are diminished and the Cu^0/1+^ peak emerges strongly due to reduction. These structural changes seem to be mainly limited to the uppermost surface and, remarkably, the Fe peaks remain virtually unaltered. Additionally, it should be emphasized that a Cu enrichment of the Fe–Cu solid solution would have resulted in a reduction in saturation magnetization, and in eventual shifts in the XRD peaks, which is shown not to be the case. Finally, it was previously established that a higher Cu content in the electrodeposited Fe–Cu films actually causes an increase in *H*_c_ rather than the reduction observed here for negative voltages.^25^ Therefore, the structural changes induced by the electric field observed in this system mainly influence the Cu species at the surface, whereas the Fe species controlling the magnetic behavior remain unaltered. Consequently, redox effects cannot be advocated to explain the voltage‐induced coercivity changes, which should then be mainly attributed to the carrier accumulation magnetoelectric effect. Indeed, iron has four unpaired 3d electrons with a high density of states near the Fermi level due to the narrow 3d band. These electrons function as the free charge carriers of the surface which govern the magnetic properties. The application of a high voltage alters the surface charge and can thereby modify the magnetic behavior, as has been demonstrated by ab initio calculations.^26^ The fluctuation in the number of 3d electrons has a direct influence on the magnetocrystalline anisotropy,^10^, ^27^ and thus on coercivity.

Finally, 5 min after removal of the voltage, a final hysteresis loop was recorded showing good recovery of the system (Figure 4c). The samples also showed good cyclability with alternating positive and negative voltage application. The corresponding *H*_c_ values during successive alternation between +21 and –21 V of applied voltage are summarized in **Table** **1**. The wait time refers to both the time between positive and negative applied voltages as well as before starting the next pair of measurements.

**Table 1:** Summary of results from cyclability measurements using the 500 nm pore sample

Wait time [min]	*H*_c_ at an applied voltage of +21 V	*H*_c_ at an applied voltage of –21 V
0	–	68
2	74	72
4	74	65
6	72	69

## 3 Conclusions

Porous, pseudo‐ordered thin metallic films consisting of nontoxic, abundant elements and prepared using a simple, easily reproducible and environmentally friendly process have been fabricated. The magnetic properties can be controlled by voltage application in a facile manner at ambient conditions leading to a reversible coercivity reduction of around 25%. According to the analysis of the film properties in the as‐fabricated state as well as after voltage application the phenomenon can be mainly ascribed to carrier accumulation/dissipation. In view of these properties, the films are good candidates for voltage control magnetic applications aimed at minimization of power consumption.

## 4 Experimental Section

*Sample Preparation, Hard‐Templating Procedure, and Electrodeposition of Porous Fe–Cu Films*: Silicon/silicon dioxide (Si/SiO_2_) substrates with a 10 nm Ti adhesion layer and a 90 nm Au seed layer and cut into 1 × 1.5 cm^2^ dimensions were used with a working area of 1 ± 0.1 cm^2^. Prior to use, the substrates were cleaned consecutively with acetone, isopropanol, and Milli‐Q water. As a preparation for electrophoretic deposition, they were treated with a 10 × 10^−3^ m solution of 3‐mercapto‐1‐propanesulfonic acid sodium salt (MPS) in ethanol at 50 °C for 1 h to increase the wettability of the surface.^28^ Finally, they were alternatingly rinsed in Milli‐Q water and ethanol to remove excessive MPS layers.

Electrophoretic deposition was implemented using a Keysight B2902A Precision Source/Measure Unit as a voltage source and a custom 3D‐printed cell consisting of a 1 cm × 1 cm × 0.6 cm poly(methyl methacrylate) (PMMA) chamber attached to a platinized titanium sheet serving as a counter electrode. Monodisperse polystyrene (PS) sub‐micrometer spheres with three different diameters (200, 350, and 500 nm) in a 2.5% w/v solid–aqueous suspension were purchased from Polysciences, Inc.

The final solution was prepared by mixing of the PS sphere suspension (0.05 mL) in ethanol (0.45 mL) and was added to the custom cell covering a 1 cm^2^ area of the substrate which was secured by slots at the back of the chamber at a 0.5 cm distance from the counter electrode. A constant potential of 40 V cm^–1^ in the case of the two smaller sphere sizes and of 60 V cm^–1^ for the largest spheres was applied with deposition times of 1 and 5 min, respectively.^28^ The deposition time was tuned to allow for either monolayer or multilayer assemblies while preserving quality regarding stability and number of defects. Following the electrophoretic deposition, the samples were promptly placed on a hot plate and heated at 50 °C for 15 min to evaporate the ethanol and stabilize the assembled PS sphere layers.^28^

Electrodeposition was carried out using a three‐electrode cell connected to a Metrohm/Eco Chemie Autolab PGSTAT302N potentiostat/galvanostat. A Pt wire served as the counter electrode and a double junction Ag|AgCl (*E* = +0.210 V/SHE) as the reference electrode. The electrolyte (100 mL) was prepared with Millipore Milli‐Q water and ACS Reagent grade chemicals purchased from Sigma‐Aldrich and contained (NH_4_)_2_Fe(SO_4_)_2_·6H_2_O (58.8 g L^−1^), CuSO_4_·5H_2_O (1.25 g L^−1^), C_6_H_11_NaO_7_ (22.9 g L^−1^), NaC_12_H_25_SO_4_ (0.2 g L^−1^), and C_7_H_5_NO_3_S (0.46 g L^−1^). The samples were deposited from the as‐prepared solution with a pH of 4.1 at a temperature of 35 °C. The optimized electrolyte composition and plating conditions were established previously with unpatterned substrates.^25^ Current densities of –25 mA cm^–2^ and deposition times between 15 and 50 s depending on the diameter of the previously deposited spheres and the desired thickness were applied. Finally, the PS spheres were removed by immersing the samples in chloroform for 3 h followed by a final rinsing in acetone, ethanol, and MQ‐water. Unpatterned, continuous Fe–Cu films of 250–300 nm in thickness were prepared whenever needed and used as control samples.

*Characterization Techniques*: The sample morphology was imaged by scanning electron microscopy using a Zeiss MERLIN FESEM. The elemental composition was determined by energy dispersive X‐ray spectroscopy (EDXS) at an acceleration voltage of 15 kV. The crystallographic structure of the deposits was studied by GIXRD using a Bruker‐AXS, model A25 D8 Discover equipped with a LinxEye XE‐T detector using CuKα radiation and a grazing incidence angle of 1°. For the surface elemental composition and oxidation state detection, XPS analyses were carried out on a PHI 5500 Multitechnique System spectrometer from Physical Electronics with a monochromatic X‐ray source placed perpendicular to the analyzer axis and calibrated using the 3d^5/2^ line of Ag. TEM analyses were performed with a Jeol‐JEM 2011 system with a field emission gun operating at 200 kV.

*Ellipsometry Measurements and Data Analysis*: In order to quantitatively account for the porosity degree of the electrodeposited Fe–Cu films, these were characterized by ellipsometry. A virtually fully dense, sputter‐deposited, Fe–Cu film of ≈100 nm thickness was prepared by co‐sputtering using an AJA International, Inc. magnetron sputtering system and was taken as a reference. Fe was sputtered at 200 W (direct current, DC) and the Cu at 20 W (radio frequency, RF) for 20 min. Variable angle spectroscopic ellipsometry and reflectivity were measured using a Sopralab GES5E rotating polarizer ellipsometer with a focused spot size of ≈250 µm and CCD detection. At least three angles of incidence were measured for each sample. The angles were adjusted to be around the Brewster angle for each sample, which decreased with pore size as one would expect for a lower refractive index. The data were analyzed using the WinElli II software. For the sputtered sample, a two semi‐infinite media approximation was applied for the direct inversion of the ellipsometric angles. The data for the electroplated samples were analyzed within the effective medium approximation, mixing the reference optical properties (those deduced for the sputtered sample) and void. Note that as the pore diameter increases, this approximation loses its validity. This was indeed observed in a worsening of the fit quality with increasing pore diameter, particularly for the data of the 500 nm pore sample. It was found that fits that assumed a homogeneous volume fraction throughout the film did not result in acceptable fits of the experimental data. The standard deviation of the fit was improved by a factor of about 5 by modeling the sample as containing two sublayers with different void content, thus strongly indicating the existence of a gradient of composition through the depth of the film, in agreement with SEM cross sections. More elaborate models, including concentration profiles or a larger number of sublayers, were also employed to analyze the data but reliable fit improvements could not be obtained due to the relatively large experimental uncertainty related to the large pore size.

*Magnetoelectric Measurements*: In‐plane hysteresis loops were recorded using a magneto‐optical Kerr effect (MOKE) setup from Durham Magneto‐Optics while applying different values of DC voltage at ambient conditions using the Keysight B2902A unit as a source. The sample was mounted vertically on a custom 3D‐printed PMMA base and placed in a quartz SUPRASIL cell filled with anhydrous propylene carbonate containing Na^+^ ions.^3^ The sodium cations served a dual function of reacting with any water entering the system to form NaOH as well as to enhance the electrical double layer owing to the presence of the dissolved Na^+^ and OH^–^ ions.^3^ The anhydrous nature of the electrolyte minimized oxidation of the Fe–Cu during the measurements. After each change of the value of applied voltage, a waiting period of 300 s was introduced before recording the hysteresis loop to allow the electrolyte to enter the pore network and the electrical double layer to form.^3^

## Conflict of Interest

The authors declare no conflict of interest.
